# Structural and Population Polymorphism of RT-Like Sequences in Avian Schistosomes *Trichobilharzia szidati* (Platyhelminthes: Digenea: Schistosomatidae)

**DOI:** 10.1155/2015/315312

**Published:** 2015-05-31

**Authors:** S. K. Semyenova, G. G. Chrisanfova, A. S. Guliaev, A. P. Yesakova, A. P. Ryskov

**Affiliations:** Institute of Gene Biology, Russian Academy of Sciences, Vavilov Street 34/5, Moscow 119334, Russia

## Abstract

Recently we developed the genus-specific markers of the avian schistosomes of the genus *Trichobilharzia*, the causative agents of human cercarial dermatitis. The 7 novel genome sequences of* T. franki, T. regenti*, and *T. szidati* revealed similarity with genome repeat region of African schistosome* Schistosoma mansoni*. In the present work we analyzed the 37 new* T. szidati* sequences to study intragenome variability and host specificity for the parasite from three localities of East Europe. DNAs were isolated from cercariae or single sporocysts obtained from 6 lymnaeid snails* Lymnaea stagnalis *and* L. palustris* from Belarus and Russia. All sequences formed three diverged groups, one of which consists of the sequences with multiple deletions; other groups involved two paralogous copies with stop codons and frameshift mutations. Strong association between geographical distribution and snail host specificity cannot be established. All studied sequences have homology with the reverse transcriptase domain (RT) of Penelope-like elements (PLE) of* S. mansoni* and* S. japonicum* and new members of RT family were identified. We proposed that three diverged groups RT sequences of* T. szidati* are results of duplication or transposition of PLE during parasite evolution. Implications of the retroelement dynamics in the life history of avian schistosomes are discussed.

## 1. Introduction

Transposable elements (TEs) are an essential part of a moderately repetitive fraction of any eukaryotic genome. Incorporating into the various regions of genome, they play a significant role in increasing mutational variability and reorganization of the genome. TEs can also alter expression of individual genes and participate in formation of the new ones [1]. Genomic rearrangements induced by TEs are often associated with a variety of adaptations to the environment [2, 3] and thus promote reproductive isolation of organisms; that is, they are implicated in speciation events [4, 5]. TEs distributions can vary among isolates of single species, so TEs have been used as markers to distinguish genetically divergent populations and subpopulations [6].

Retrotransposons constitute a significant proportion of the TEs; their typical characteristic is the use of the reverse transcription mechanisms involving a reverse transcriptase (RT). Retrotransposons are major components of eukaryotic genomes and have been usually divided into four classes containing the long terminal repeat (LTR+), without LTR (non-LTR), Penelope-like elements (PLE), and DIRS. Non-LTRs are grouped into 11 different clades, based on the phylogeny of RT domains [7].

Several retrotransposons have been identified in blood flukes of the genus* Schistosoma*. Despite the fact that they are more than half (58.5%) of the repetitive elements [8, 9], detailed characteristics are known only for a few LTR retrotransposons (Boudicca, Gulliver for* S. mansoni* and Tiao for* S. japonicum*), as well as for some non-LTR retrotransposons (CR1 for* S. mansoni* and* S. japonicum* and SjR2 and Pido for* S. japonicum*) [10]. The third class of retrotransposons, PLE, is widespread among eukaryotes including schistosomes in which only two of PLEs were described [11, 12].

As compared with these* Schistosoma* species, the genome of our object belongs to a more ancient group of the blood flukes infecting waterfowl, namely,* Trichobilharzia*, and is still virtually unexplored.

We converted RAPD amplicons into SCAR (Sequence Characterized Amplified Region) markers for three avian schistosome species* T. szidati*,* T. franki,* and* T. regenti* and found new genus-specific repetitive sequences which revealed 64% homology with the repeat region of* Schistosoma mansoni* [13]. For that reason, a pair of specific primers TR98F and TR98R was matched to the sequence of one* T. franki* RAPD spectrum amplicon. Following PCR allowed us to detect 391–393 bp fragments in the spectrum of each species and additional shorter fragment 274 bp was amplified only in* T. szidati* which parasitized one snail* Lymnaea stagnalis*. A few species-specific mutations and indels were found in seven nucleotide sequences from three schistosome genomes studied and confirm the suitability of these sequences for molecular diagnostics of species of genus* Trichobilharzia* [13].

In another study we assessed the overall representation of different types of repeats in a small RAPD library of* T. szidati* obtained from clonal offsprings, individual cercariae within daughter sporocysts. 50 polymorphic nonoverlapping DNA fragments ~300–1500 bp were revealed from RAPD patterns of 47 individual genomes of parasites infected 6 freshwater snails* L. stagnalis*. These sequences contained tandem, inverted, and dispersed repeats as well as regions homological to retroelements of two human parasites,* S. mansoni* and* S. japonicum*. Tandem and inverted repeats constituted 8.9% and 22.1%, respectively, while the percentage of dispersed repeats was 21.0%. About 40% of sequences of approximately 1000 bp included regions which displayed amino acid homology with open reading frame* pol* products of* S. mansoni *and* S. japonicum *retroelements: nonlong terminal repeat retrotransposons (nLTRs, 76%), long terminal repeat retrotransposons (LTRs, 14%), and Penelope-like elements (PLEs, 10%). Most of these regions (86.4%) contained frameshifts, gaps, and stop codons [14]. In the present study one of the SCARs is to provide detailed characteristics of* T. szidati* intragenome variability for the first time. Furthermore we examined the host specificity of the parasites from three geographic localities obtained from two freshwater snail species* L. stagnalis* and* L. palustris*. We present the results of structural, phylogenetic, and bioinformatic analyses to determine the distribution and possible functions of 37 newly identified genomic sequences belonging to the RT domain as a part of the PLE in* T. szidati*. We also demonstratedfor the first time that* T. szidati *genomes contain threedivergedgroups ofRT sequences which are result of duplication or transposition of TEs during parasite evolution.

## 2. Material and Methods

### 2.1. Collection Sites and Sequence Generation

A total of 6* T. szidati* isolates (infrapopulations) were collected from the freshwater snails* Lymnaea stagnalis (Ls, n* = 3) and* Lymnaea (Stagnicola) palustris (Lp, n* = 3). The snails were sampled from the three geographical localities, Moscow freshwater pond Altufyevo (in 2005), Lake Naroch (Belarus, 2008), and Lake Onega (Karelia, Russia, 2012) (Table 1). Total genomic DNA was extracted from 5–10 ethanols fixed free-swimming mature cercariae or fragments of individual sporocysts as described previously [14]. PCR with a specific primer pair, TR98F (CTCCGACTGATGATGACAAGAAGA) and TR98R (ATGAGTGGCGAACGGTATCCT), and cloning and sequencing of amplified products were carried out as described [13]. For each PCR fragment 2–5 clones were sequenced. In total, 37 newly generated sequences were analyzed, of which 30 clones contained inserts of 390 or 391 bp and 7 contained the shorter inserts of 274 bp in size. All sequences were deposited in GenBank under accession numbers KP889985-890021.

### 2.2. Data Analysis

Multiple alignments were made with CLUSTAL and MUSCLE algorithms implemented in MEGA5.2 [15] software and were edited manually. Search of stop codons in alignments, AT/GC ratio, mean pairwise genetic distances (min-max, overall *d*-distance) [16], and codon based *Z*-test of neutrality (Nei-Gojobori method with Jukes-Cantor correction and 1000 bootstrap replications) were made using MEGA version 5.2. Phylogenetic analysis (Neighbour Joining and Bayesian Inference) was performed by MEGA version 5.2. and MrBayes version 3.2.2. software [17]. The best-fit nucleotide substitution model was selected using jModelTest version 2.1.6. [18]. We used HKY model for Bayesian analysis with two simultaneous runs of four chains for 5 000 000 generations, sampling trees every 500 generations. The first 25% trees sampled were discarded as “burn-in.” For comparative analysis we used three sequences of* T. szidati* deposited in GenBank (Acc. numbers GU980751–GU980753, [13]).

Similarity searches of homology between our nucleotide sequences of* T. szidati* and previously known nucleotide and amino-acid sequences of mammalian schistosomes and other trematodes have been performed using BLAST (*blastn*,* blastx,* and* tblastx*) with the default parameters [19].

## 3. Results

### 3.1. Analysis of Intra- and among Population Variability

Six DNA patterns were obtained with the use of two specific primers TR98F and TR98R and DNA templates isolated from mature cercariae or individual sporocysts from six isolates infecting the three snails of* L. stagnalis *(*Ls*) and three snails of* L. palustris *(*Lp*) during the course of PCR amplification. Each of the patterns comprises two amplicons with the identical intensity of UV luminescence and approximate size of 400 and 300 bp. The size of the cloned sequences of the longer amplicon (*n* = 30) reached 390–391 bp, and the sequences of the shorter fragment (*n* = 7) contained 274 bp. Only 17 sequences out of 40 were unique, and the rest contained from two to four identical copies.

Estimates of genetic heterogeneity of each of the six* T. szidati* infrapopulations (isolates from single snails) are presented in Table 1. They were obtained by calculation of genetic distances for each pair of sequences of the size 390 and 391 bp of free-swimming mature cercariae (isolates Sz3 and Sz11) and fragments of single sporocysts (isolates Sz12 and Sz43). The maximum and minimum estimates of divergence between pairs ranged from 0 to 21.1% and depend mainly on the size of the sample. Sequence divergence was revealed to be up to 0.3% in a few samples of the parasite of snails* Ls*. The maximum differing copies (up to 21%) were found among the parasites that infect the snails* Lp* (isolates Sz11, Sz12, and Sz43). Despite this, the average levels of divergence between the copies do not differ much for cercariae isolated from the two different species of snails (10.5% and 11.3%). In the total sample, the divergence of copies reaches 24.1%.

**Figure 1:**
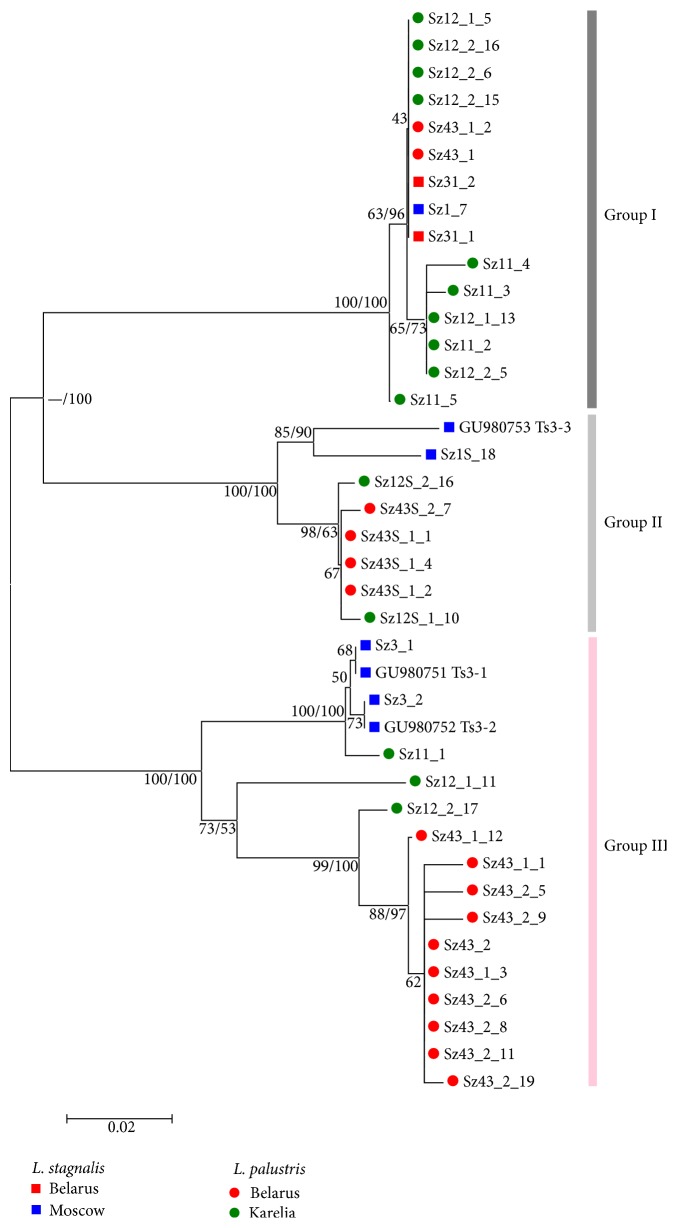
Phylogenetic tree of* T. szidati,* inferred from 40 RT-like sequences. Topology was inferred using MEGA 5.2 software (NJ, p-distance, 1000 bootstrap replications) and confirmed by MrBayes 3.2.2. Node support values are shown as follows: the first value is Bayesian posterior probability assessed using MrBayes software, and the second value is bootstrap support assessed by NJ method using MEGA 5.2 software. Sequences belonging to different localities and host snails are shown by differently colored figures (see the legend).

The reason for such a high intraspecific heterogeneity becomes apparent in the construction of the dendrogram of genetic differences, demonstrating the distribution of 40 sequences in six infrapopulations from the three geographical localities (Figure 1). 15 sequences of 390 bp in size (Group I) and 17 sequences of 391 bp (Group III) are combined in two large clusters with high reliability (IB = 100%). Thus, the full-length amplicons form the two groups of significantly diverged sequences.

The intragroup differentiation is somewhat higher for Group III (*D* = 3.9%) compared with Group I (*D* = 0.6%), whereas the intergroup differences account for 24.1% (Table 2). Eight sequences of short copies of 274 bp form its own cluster (IB = 100%). It is composed of two distinct copies of the isolate Sz1 derived from the* L. stagnalis* Ls1 (Moscow). Apart from them, there are six sequences of the two schistosome isolates Sz12 and Sz43 from snails* L. palustris* (Karelia and Belarus) (Figure 1, Group II). The average value of *D* in the group II is 2.2%. In Group I, we found no clear subclusters neither characterizing geographic population identity nor belonging to either of two species of intermediate snail hosts. In the third group, four sequences of the parasite from one* L. stagnalis* from Moscow (isolate Sz3) and schistosome sequences from* L. palustris* from Belarus (isolate Sz43) comprised their own subclusters. The sequences of two isolates Sz11 and Sz12 from Karelian mollusks* L. palustris* either fall into one of the two subclusters or stand quite separately (e.g., variant Sz12_1_11 on Figure 1). Note that another sequence of isolate Sz11, namely, Sz11_5, holds an isolated position in Group I.

Occasionally, snails in natural populations can be infected with not one but two or more miracidia having different genotypes. This leads to biased estimates of variability in some infrapopulations. Therefore, we compared the variability not only of mature cercariae but also of individual sporocysts. For this purpose, two sporocysts (spc1 and spc2) were isolated from each of the two snails* L. palustris* from Belarus (Sz43) and Karelia (Sz12) and for each of them from four to seven sequences were obtained. Individual variability of sporocysts consisted of the presence of two or three differing copies in each of the three groups of sequences.

Sequences of Groups I and II were simultaneously identified in only three of sporocysts, where the average level of divergence was high and reached 8.1% (Sz12_2), 10.6% (Sz43_1), and 13.7% (Sz12_1). All sequences of the remaining sporocyst Sz43_2 belong to Group II and were almost identical (*D* = 0.5%). All short copies were also almost the same, both within individual sporocysts and between sporocysts from the same mollusk (Figure 1), while the most divergent two copies from Group II (Sz12S_2_16 and Sz12S1_10) and Group III (Sz12_1_11 and Sz12_2_17), which define the highest level of infrapopulation variability in Sz12, are the part of the genomes of both sporocysts 1 and 2 of mollusk Sz12. Thus, comparing the genetic heterogeneity of cercariae from one sporocyst, we demonstrated that the composition of a bird schistosome* T. szidati* genome could simultaneously present three groups of copies of closely related sequences. Maximum intragenomic divergence is typical for the parasite infrapopulation from mollusk* L. palustris* (Karelia) and can reach 20%.

The distribution of copies in the six infrapopulations of snails indicates a lack of host specificity. However, there is a tendency to the formation of specific sets of copies of Groups II and III in geographically isolated parasite infrapopulations* L. stagnalis* from Moscow and in the pooled sample of parasites of* L. palustris* from Karelia and Belarus. However, to clarify these results, it is necessary to study more representative samples of* Trichobilharzia*–infected snails from wider geographical origins.

### 3.2. Analysis of the Sequence Structure and Search Similarity

**Figure 2:**
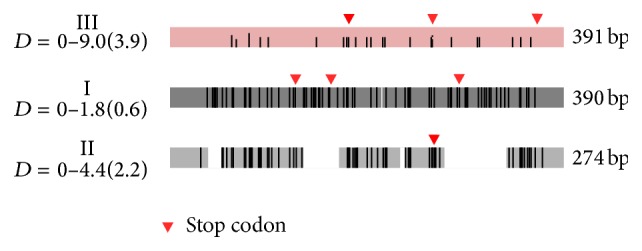
Schematic image of the distribution of polymorphic sites and deletions and the location of stop codons in each of the three RT-like sequences groups of* T. szidati*. D: min-max and average (in parentheses) pairwise genetic distances.

Figure 2 gives schematically the distribution of polymorphic sites, deletions, and the location of stop codons in each of the three groups of sequences. All sequences of Group I have a length of 390 bp due to a single nucleotide deletion at position 211 compared with the sequences of Group III (391 bp). All short sequences of Group II with the size of 274 bp have four extended deletions of total length 117 bp (14 nt: 38–51 bp, 36 nt.: 133–168 bp 5 nt.: 229–233 bp, 62 nt.: 273–334 bp). Groups I and III sequences each contains three stop codons and Group II, one stop codon. Their position is indicated with triangle in Figure 2. The second stop codon in Group III is revealed only in a half of the sequences. An excess of AT-bases (AT : GC = 60.4 : 39.6) known in all trematodes is found in the nucleotide composition of all sequences (Table 2). Average estimates of divergence of nucleotide and amino acid sequences are the same for Groups II and III, and amino acid divergence is somewhat higher comparing to nucleotide divergence in Group I (Dn = 0.6, Da = 1.1). Codon-based *z*-test of neutrality showed that observed mutations are significantly neutral only within concatenated Groups I and III. Within each group separately results of neutrality test are insignificant, as well as results of both purifying and positive selection tests (Table 2).

Table 2 summarizes also the results of* T. szidati* sequence homology with nucleotide and amino acid sequences available throughout the NCBI. We found no significant similarity of* T. szidati* nucleotide sequences in Groups I and II with extended sequences of mammalian schistosome genomes (blastn). Only for Group III, a high similarity of short segments of the sequences (about 40 nt) with the intergenic regions of 1, 2, 3, 4, and 7 and W-chromosomes of* S. mansoni* as well as with more extensive (about 70 nt) unannotated region of the BAC-clone of* S. japonicum* (C108_113I17, FN293021) was revealed.

Translated nucleotide sequence similarity search (blastx) revealed a significant homology between the sequences of Groups I and III and the reverse transcriptase domain of two mammalian schistosome species,* S. mansoni* (Ass.n. SA00247) and* S. japonicum* (Ass.n. SAX83715). The length of these GenBank sequences is equal to 394 and 500 amino acid residues, respectively, and the regions of similarity are located almost at the 5′ terminus and comprise about 125 amino acid residues of the mammalian schistosome's RT. Moreover, a significant similarity (41–51%) with the two shorter regions (30–80 aa) of the RT sequence of the human fluke* Clonorchis sinensis* (532 amino acid residues, GAA47523) was found. Therefore, the results of similarity search with known* Schistosoma* amino acid sequences indicate that new sequences of* T. szidati* belong to the family of reverse transcriptase (RT) genes.

Furthermore, using another BLAST algorithm (blastx) the highest similarity our sequences (~36% for Group III, ~42% for Group I) was found to the 5′ terminus of the reverse transcriptase domain of two retrotransposons of* S. mansoni* (Perere-10, BN000801) and* S. japonicum* (Sj-penelope2, FN356226), belonging to a large class of Penelope-like transposable elements. In addition, the amino acid sequences of Group III demonstrate 27–47% similarity with the short regions on the chromosomes 1–4, 7 and W-chromosome of* S. mansoni*, included in the introns of hypothetical proteins or noncoding intergenic regions.

## 4. Discussion

Search for homology with known schistosome amino acid sequences of the Schistosoma genus indicates that new genome sequences of the avian schistosomes* T. szidati* belong to the RT domain, which is common to all retroretroelements. Reverse transcriptase is the most highly conserved protein encoded by retroviruses and retrotransposons. This peculiarity allows the use of RT sequences as recognizing phylogenetic signature of host taxa in a retrotransposon phylogeny, besides that, for studying the dynamics of retroposition in the life cycle to determine its life history [20, 21].

Relatively little is known about intraspecies and intragenomic variability of RT among invertebrates. Usually, it does not exceed 10% for the members of the same subfamily.

Thus, mean intraspecific divergence is 2.88% between reverse transcriptase sequences of SURL elements (from the* gypsy* group) in the closely related echinoid species* Strongylocentrotus purpuratus* and S.* droebachiensis* [22]. Several families of elements were found in African and Asian schistosomes, which were characterized by more than 80% of similarity in amino acid sequences of RT. It has been shown that a family can combine both copies of the same element and the closely related elements [23].

Significant variability in the composition of RT-like sequences (0–21.2%) of 390-391 bp in size was found when studying* T. szidati*, infecting three snails of* L. palustris*. These estimates do not depend on the geographical location of the snails (Belarus and Karelia), nor on the stage of the parasite life cycle (free-swimming mature cercariae or fragments of single sporocysts).

The main reason for the high heterogeneity of the RT-like sequences of* T. szidati* is a simultaneous occurrence of significantly diverged copies of Groups I and III in one genome (Table 1, Figure 1).

The nucleotide and amino acid divergence between RT copies of these groups is 20% on average reaching the level of 45% for individual copies (Table 2). Given the lack of detailed annotation of the complete genome sequences of African and Asian mammalian schistosomes, we are able to conditionally include all detected RT copies to the members of the same family for the present. The average sequence divergence in a group is less than 4%; thus presumably we are dealing with representatives of several RT lines or subfamilies.

All detected RT-like copies probably are inactive copies as they contain either stop codons (Groups I and III) or a single nucleotide deletion (Group I), modifying the reading frame (frame shift mutation). Note that short copies of 274 bp in size of Group II pertain to inactive copies.

These copies with typical extended deletions are more degenerated comparing with the previously mentioned RT copies. Due to the fact that any detected changes in the structure of RT sequences in* T. szidati* are incapable of coding for a functional reverse transcriptase (breaking the reading frame of RT), we can refer the elements of each of the three groups to pseudogenes, derived from the RT protein-coding gene. Since the pseudogene evolved under neutrality (*Z*-tests, Table 2), they may show the higher level of diversity in some cases. For instance, there is a 45% of sequences divergence between the pairs Sz43_2_10 and Sz12_1_5 (Figure 1, Table 1). Thus, for the first time, we have discovered the three types of degenerated RT copies in the same genome of avian schistosomes probably belonging to a few closely related subfamilies of transposable elements.

To date, from all deposited RT-containing retrotransposons of mammalian schistosomes (see Introduction) new obtained sequences detected in the* T. szidati* genome show significant similarity with representatives of the Penelope-like elements (Perere-10 and Sj-penelope2). Therefore, we have reason to include currently all identified RT copies in the* T. szidati* genome to a class of PLE.

The absence of intact sequences among the discovered copies indicates their ancient origin, while the older group seems to be a group of highly degenerated and reduced in size sequences of Group II. Compared with them, paralogs of Group I are less degenerated, having only one reading frame shift mutation and several stop codons. Copies of Group III with two or three stop codons degenerated probably much later, and therefore only for copies of this group, small areas of similar genomic sequences on the five chromosomes of mammalian schistosome* S. mansoni* were found as well. Besides, their use for phylogenetic reconstruction demonstrates the presence of intraspecies structure in* T. szidati* (Figure 1).

We cannot yet reconstruct a detailed scenario for the origin and invading of discovered RT-like sequences in populations of* T. szidati* on the limited material. It is likely that the occurrence of paralogous RT copies is associated with transposition bursts that took place in the remote past of avian schistosomes. Apparently, two acts of transposition bursts could result in the three types of RT copies in the genome of modern* T. szidati*. The most compelling evidence of this assumption will be obtained from the analysis of the whole genome sequencing of different species of avian schistosomes. Currently, similar analysis was carried out for genomes of the schistosomes* S. mansoni* and* S. japonicum* [23]. Considerable differences in retrotransposon representation have been shown between the two species (22% and 13%, resp.). A large part of this difference can be attributed to higher representation of two previously described retrotransposon families SR2 and Perere-3/SR3 of* S. mansoni*. It was suggested that the* S. mansoni* SR2 families were the subject of recent bursts of transposition that were not paralleled by their* S. japonicum* counterparts. It was hypothesized that these bursts could be a consequence of the evolutionary pressure resulting from migration of* Schistosoma* from Asia to Africa and their establishment in this new environment, helping both speciation and adaptation [23].

Similar processes could occur during the life history of avian schistosomes. Their definitive hosts, ducks of the family Anatidae, are characterized by long-distance spatial and temporal migrations, changing of ecological niches, and multiple range expansions [24]. It is necessary to add that processes of snail-parasite interactions, occurring during the development or change of the intermediate snail host, also have a significant role in the genetic differentiation of schistosomes [25]. During the evolutionary radiation of mammalian schistosomes, Asian and African groups have adapted to parasitizing on the snails of different groups of Gastropoda. African schistosomes infected representatives of several families of pulmonate snails (Pulmonata) and Asian species feed on mollusks belonging to the Caenogastropoda. These processes resulted just in mitochondrial genome rearrangement. Thus, the ancestral gene order of mtDNA is conserved amongst East Asian* Schistosoma* spp. [26] and different amongst species sampled from Africa or India [27, 28]. In the evolution of a more ancient group of schistosomes, namely, avian schistosomes, multiple repeated changes of the definitive and intermediate hosts could also occur as well as generating of new molecular adaptations, and increasing the transposition activity of TEs may serve as markers of such events.

## 5. Conclusions

In the present work 37 new sequences obtained from genome of avian schistosome* Trichobilharzia szidati* parasitized 6 lymnaeid snails* L. stagnalis* and* L. palustris* from Belarus and Russia were revealed. Phylogenetic reconstructions and BLAST search results indicate that all studied sequences demonstrate homology with the reverse transcriptase domain (RT) of Penelope-like elements of African and Asian mammalian schistosomes* S. mansoni* and* S. japonicum*. Future whole genome sequencing and population-wide analysis of avian schistosomes will help to understand the features of the retrotransposon expansion during host-parasite coevolution.

## Figures and Tables

**Table 1:** General data for 6 cercarial isolates from *L. stagnalis* and *L. palustris* and for 40 sequences obtained by the primers TR98F and TR98R. Distribution of clones examined between three groups indicated in Figure 1 is shown in square brackets [I, II, III].

Isolate	Host	Locality	Total number of sequences		^**^ *D*-distance (%), min–max (average)
			Cercariae	Sporocysts	
Sz31	*Ls *	Belarus	2 [2, 0, 0]		

				spc 1 [1, 3, 3]	0.3–21.1 (10.6)
Sz43	*Lp *	Belarus	2 [1, 0, 1]	spc **2 [**0**, 1, 6]**	**0–1.3 (0.5)**
				Σ = 14 [1, 4, 9]	0–20.9 (6.5)

Sz11	*Lp *	Karelia	5 [4, 0, 1]	—	0.5–19.6 (8.6)

				spc 1 [2, 1, 1]	0–20.1 (13.7)
Sz12	*Lp *	Karelia	—	spc **2 [**4**, 1, 1]**	**0.3–19.9 (8.1)**
				Σ = 10 [6, 2, 2]	0–20.5 (9.1)

Sz1	*Ls *	Moscow	2 [1, 1, 0]	—	—

Sz3^*^	*Ls *	Moscow	5 [0, 1, 4]	—	0–0.3 (0.2)

Σ	*Ls *		9 [3, 2, 4]	—	0–18.3 (10.5)
	*Lp *		7 [5, 0, 2]	24 [7, 6, 11]	0–21.2 (11.3)
	*Ls* + *Lp *		16 [8, 2, 6]	24 [7, 6, 11]	0–45.9 (24.1)

^*^This group includes three previously deposited sequences GU980751–GU980753. ^**^The comparison was carried out only for 390 and 391 bp fragments; spc: sporocyst.

**Table 2:** Characteristics of intra- and intergroup polymorphism and results of similarity search of studied *T. szidati* sequences.

		Similarity		
Groups	Polymorphism	BLASTN	BLASTX	TBLASTX
		(+/+)	(Frame +3)	(Frame +3/+3)
Group I	*N* = 15 *L* = 390 *V* = 12 Pinfo = 2 AT : GC = 62.3 : 37.7 *Z* test: dN-dS = −0.021 (*P* = 0.983) Dn = 0.6 Da = 1.1	No	Sm RT CAJ00247: score 49.3–52.4 bits, Exp 2*e* − 05–2*e* − 04, cover 51% (15–215 bp), **I = 39–42%** Sj RT CAX83715: score 48.1–50.8 bits, Exp 6*e* − 05–1*e* − 04, cover 54% (3–215 bp), **I = 35–38%** Cs RTGAA47523: score 45.8–46.2 bits, Exp 0.002–0.003, cover 40% (57–215 bp), **I = 42%**	Sj Penelope-like element retrotransposon Sj-penelope2 FN356226: score 65.4–70.2 bits, Exp 3*e* − 24–4*e* − 17, cover 98% (3–215 bp), **I = 39**–**42%** Sm Penelope-like retrotransposon Perere-10 BN000801: Score 54.2–58.8 bits, Exp 3*e* − 13–6*e* − 06, Cover 53-54% (15–221 bp), **I = 39**–**42%**

Group II	*N* = 8 *L* = 274 *V* = 20 Pinfo = 20 AT : GC = 57.8 : 42.2 *Z* test: dN-dS = −0.949 (*P* = 0.344) Dn = 2.2 Da = 2.0	No	Cs RTGAA47523: score 35.0, Exp 4.5–4.6, cover 43–48 (466–521 bp), **I = 38-39%**	No

Group III	*N* = 17 *L* = 391 *V* = 24 Pinfo = 85 AT : GC = 58.7 : 41.3 *Z* test: dN-dS = −1.597 (*P* = 0.113) Dn = 3.9 Da = 3.9	S j Anhui clone BAC C108_113I17 FN293021: score 55.4 bits Exp *e* − 0.4; cover 17% (308–375 bp); **I = 78%** Chr1, chr2, chr3, chr4, chr7, Wchr: score 48.2–46.4 bits Exp *e* − 0.26; cover 15–19% (116–220 bp); **I = 70–77%**	Sm RT CAJ00247: score 64.7–73.6 bits, Exp 9*e* − 13–2*e* − 07, cover 91–95% (15–386 bp), **I = 30–37%** Sj RT CAX83715: score 50.4–60.8 bits, Exp 9*e* − 05–2*e* − 07, cover 77–95% (3–374 bp), **I = 30–32%** Cs RTGAA47523: score 46.2–52.2 bits, Exp 5*e* − 05–0.002, cover 39–41% (3–374 bp), **I = 47–49%**	Sj Penelope-like element retrotransposon Sj-penelope2 FN356226: score 70.2–103.0 bits, Exp 1*e* − 26–2*e* − 19, cover 98% (3–386 bp), **I = 34–36%** Sm Penelope-like retrotransposon Perere-10 BN000801: score 82.6–83.1 bits, Exp 2*e* − 20–3*e* − 13, cover 95% (15–386 bp), **I = 35%** Sm Chr1, chr3, chr4, chr7, Wchr: score 118–48.2 bits, Exp *e* − 14–*e*28; cover 98-99% (3–386 bp), **I = 27–47%** S j Anhui clone BAC C108_113I17 FN293021: score 35–49.2 bits, Exp 1*e* − 06–4*e* − 04; cover 40–93 (81–230 bp); **I = 26–40%**

Groups I + III	*N* = 32 *L* = 391 *V* = 203 AT : GC = 60.4 : 39.6 Pinfo = 182 *Z* test: dN-dS = −2.640 (*P* = 0.009) Dn = 24.1 Da = 21.6	—	—	—

*N*: the sequence numbers, *L*: length (bp), *V*: the number of variable sites, Pinfo: the number of parsimonial sites, *D*: distance (%), Sm: *S. mansoni*, Sj: *S. japonicum*, Cs: *Clonorchis sinensis*, RT: reverse transcriptase, Dn: nucleotide divergence, Da: amino acid divergence.
